# Supplementary material for: Exploration of the Possible Relationships Between Gut and Hypothalamic Inflammation and Allopregnanolone: Preclinical Findings in a Post-Finasteride Rat Model
Source: Biomolecules. 2025 Jul 18;15(7):1044. doi: 10.3390/biom15071044 (PMC12293867; doi:10.3390/biom15071044)
Supplement: Supplementary file 1 [file biomolecules-15-01044-s001.zip › biomolecules-3641773/biomolecules-3641773-supplementary.docx]

**Supplemental Material**

**Supplementary S1: Processing of Intestinal Tissue and Macrophage Isolation**

Intestinal tissues were harvested and processed to remove the epithelial layer as reported [20]. Briefly, the samples were incubated in Ca^2+/^Mg^2+^ free HBSS with 5% FBS (HBSS/FBS) (ThermoFisher scientific,) containing 2 mM EDTA (ThermoFisher scientific) at 37°C for 20 minutes with horizontal shaking. After incubation, the solution containing epithelial cells was discarded, and tissues were rinsed with fresh HBSS/FBS with EDTA. The remaining tissues were dried on absorbent paper, minced using sterilized scissors, and transferred to tubes containing HBSS/FBS supplemented with collagenase (1.5 mg/mL, Merk Life Science S.r.l.) and DNase enzymes (0.04 mg/mL, Roche). Tubes were incubated at 37°C for 20 minutes with horizontal shaking and vortexed. Digested tissue was filtered through a 70 μm metal mesh using a syringe plunger, washed with cold HBSS/FBS, and centrifuged at 4°C at 1500 rpm for 5 minutes. The pellets were washed twice with cold HBSS/FBS and resuspended in cold PBS.

**Flow Cytometry Analyses**

For flow cytometry analyses, pellets were first resuspended in Live-Dead viability/cytotoxicity staining solution (prepared according to manufacturer instructions, ThermoFisher scientific) and incubated at 4°C for 30 minutes. After washing with cold PBS, cells were fixed in 4% PFA (Merk Life Science S.r.l.) for 15 minutes at room temperature, washed with PBS, and stored at 4°C until further processing.

Fixed cells were blocked in rat serum (ThermoFisher scientific) at 4°C for 10 minutes, followed by centrifugation and resuspension in a cocktail of primary antibodies, including anti-CD45, anti-CD11b, anti-CD68 (ThermoFisher scientific) and anti-CD86 (BD Bioscience). After a final centrifugation step, the cells were resuspended in PBS and transferred to flow cytometry tubes for analysis. The analyses were performed using Fortessa BD X20 (BD Biosciences).

**Supplementary S2: SCFAs Quantification and Analysis**

The absolute quantification of SCFAs (i.e., acetic, propionic, iso-butyric, butyric, valeric and iso-valeric acids) by LC-MS/MS was assessed by LC-MS/MS in stool samples as previously described [23], with some modifications. Briefly, 20 mg of samples were extracted in 1 mL of a 1:1 v/v water (H2O)/acetonitrile (ACN) solution. The mixtures were homogenized using a Tissue Lyser for 1 minute at 30Hz. Subsequently, samples were centrifuged at 4°C and 18000xg for 10 minutes. The supernatant was carefully transferred into a 1.5 mL eppendorf tube, diluted 1:200 and 20 μL of internal standard (butyric-1,2-13C2 acid 50 ng\μL) were added to each sample. 50 μL of the sample were derivatized by adding 2.5 μL of a 2.4 M aniline solution (in ACN), followed by 2.5 μL of a 1.2 M N-(3-Dimethylaminopropyl)-N′-ethylcarbodiimide hydrochloride solution (in H_2_O). The reaction mixture was kept on ice for 2 hours, with regular mixing, following this an aliquot was diluted 1:20 in H_2_O/ACN (1:1 v/v) and submitted to LC-MS/MS analysis. For the quantitative analysis 5μL/sample were injected in API 3500 (AB Sciex, USA) mass spectrometer, equipped with an electrospray source (ESI+) and triple quadrupole analyzer, interfaced with a pump for the HPLC model EXION SL (Sciex, USA). The chromatographic separation was achieved using Luna Omega 5 μm PS C18 100 Å column (Phenomenex, USA). The mobile phases consisted of 0.1% formic acid in water and 0.1% formic acid in methanol at flow rate of 0.300 μL/min. The quantitative analysis was performed on the basis of calibration curves freshly prepared. LC–MS/MS data were evaluated using Analyst software (Sciex, USA).

**Supplementary S3: Metabolomics Analysis Methods**

**Metabolomic Profiling**

Cecal samples underwent an untargeted metabolomic analysis involving extraction, purification, and derivatization processes utilizing the MetaboPrep GC kit (Theoreo srl, Montecorvino Pugliano, Italy), following the protocols established by Troisi et al. [71, 72]. In summary, 25 mg of sample were aliquoted into Eppendorf tubes and mixed with an alcohol-based extraction reagent containing 2-isopropyl malic acid as an internal standard. The mixtures were vortexed at 1250 rpm for 30 minutes and subsequently centrifuged at 16,000 rpm for 5 minutes at 4 °C. From the supernatant, 200 µL was transferred to new tubes, subjected to another vortexing at 1250 rpm for 30 seconds with a purification solution, and centrifuged again under the same conditions. A total of 175 µL of the resulting supernatant was placed into glass vials and freeze-dried overnight. Derivatization proceeded in two stages: initially, a methoxylamine pyridine solution was introduced and the mixtures were vortexed at 1200 rpm for 90 minutes. Following this, 25 µL of N,O-Bis(trimethylsilyl)trifluoroacetamide (BSTFA) derivatizing agent was added, and the vials were further vortexed at 1200 rpm for an additional 90 minutes.

The derivatized metabolites were then transferred to gas chromatography (GC) vials equipped with low-volume inserts suitable for autosampler injection. Prior to GC-MS analysis, the vials were centrifuged at 16,000 rpm for 5 minutes while maintaining a temperature below 4 °C.

For chromatographic separation, 2 µL of each derivatized sample was injected into a GCMS-2010SE system (Shimadzu Corp., Kyoto, Japan). Separation was achieved on a CP-Sil 8 CB fused silica capillary column (30 m × 0.25 mm, 1.00 µm film thickness; Agilent, J&W). Helium served as the carrier gas with an initial oven temperature of 100 °C held for 1 minute, then ramped to 320 °C at a rate of 6 °C per minute, followed by a hold at 320 °C for 2.33 minutes. The carrier gas flow was adjusted to a constant linear velocity of 39 cm/s with a split ratio of 1:5. Detection was performed using electron impact ionization (70 eV) in full scan mode (35–600 m/z), with a scanning speed of 3333 amu/sec and a solvent delay of 5 minutes.

**Metabolite Identification**

Metabolite identification was conducted in accordance with the protocols outlined by Troisi et al. [73]. To characterize untargeted metabolites, each chromatographic peak’s mass spectrum was analyzed and compared against the comprehensive NIST-2014 library available at the National Institute of Standards and Technology (NIST) facility in Gaithersburg, MD, USA. This comparative analysis employed a maximum tolerance of 10 units for the differential linear retention index. Additionally, spectral matching within the library was restricted to a minimum similarity threshold of 85%, adhering to the stringent Level 2 identification criteria as defined by the Metabolomics Standards Initiative (MSI) [73, 74]. Metabolites that did not fulfill these criteria were designated as “unknown,” in strict compliance with MSI Level 4 standards.

Chromatographic-mass spectrometric signals were excluded from subsequent analyses if they met any of the following conditions: absence in more than 20% of the samples, insufficient concentration levels, or suboptimal spectral quality that hindered reliable metabolite identification.

Through this rigorous identification process, a total of 248 endogenous metabolites were confidently identified. To ensure the statistical significance of these metabolites in discriminating between different classes or categories, only those with Variable Importance in Projection (VIP) scores exceeding 1.5 were selected for further validation. Confirmation of these high-VIP metabolites was performed using independent analytical standards, in alignment with the rigorous Level 1 identification criteria established by the MSI.

**Comprehensive Metabolomic Data Analysis**

The metabolomic data derived from GC-MS were consolidated into a comma-separated matrix file, which was subsequently imported into MetaboPredict® software (Theoreo S.r.l., Montecorvino Pugliano, Italy) for subsequent statistical evaluations. Prior to analysis, the chromatographic data underwent a series of preprocessing steps to ensure data quality and consistency. Data alignment was achieved using a parametric time warping algorithm [74], followed by essential procedures including peak detection, integration, and deconvolution. The processed chromatographic data were organized into a structured table, with each row representing an individual sample and each column corresponding to a specific metabolite.

To enhance data reliability, multiple normalization techniques were applied. This included a generalized logarithmic transformation for data transformation and an auto-scaling method for data scaling, which involved mean-centering and scaling based on the standard deviation of each metabolite. Additionally, normalization accounted for the chromatographic peak area of the internal standard and the precise weight of each sample [73].

Class separation was further investigated using Partial Least Squares Discriminant Analysis (PLS-DA), a supervised multivariate regression method that identifies linear combinations of the original variables (X) capable of predicting class membership (Y). To evaluate the significance of the class discrimination, permutation testing was performed. In each permutation, a PLS-DA model was constructed using the original data (X) and randomly shuffled class labels (Y), while determining the optimal number of components through cross-validation based on the true class assignments. Two statistical measures were employed to assess class discrimination: prediction accuracy during model training and the separation distance, quantified as the ratio of the sum of squares between groups to the sum of squares within groups (B/W ratio). If the observed test statistics fell within the distribution generated from the permuted class labels, the class discrimination was considered statistically insignificant [71].

Furthermore, Variable Importance in Projection (VIP) scores were calculated for each component within the PLS-DA model. The VIP scores represent a weighted sum of the squared PLS loadings, reflecting the contribution of each metabolite to the explained variation in Y across the different dimensions.

**Pathway Analysis**

Pathway analysis was conducted by integrating pathway enrichment results with pathway topology assessments, following the methodology of Xia and Wishart [75] . The Kyoto Encyclopedia of Genes and Genomes (KEGG) metabolic pathways served as the reference database within the MetPa algorithm-based web application.

Initially, over-representation analysis was performed to determine whether specific pathways contained a higher number of altered metabolites than expected by chance. This was evaluated using the hypergeometric test to compare the observed number of metabolites within each pathway against a random distribution.

To incorporate the structural aspects of metabolic networks, pathway topology analysis was executed using betweenness centrality, a metric that quantifies the importance of each node (metabolite) within the network. This analysis provided insights into the key metabolites that influence pathway functionality and interconnectivity.

Given the multiple pathways evaluated simultaneously, p-values from the enrichment analysis were adjusted for multiple comparisons using the False Discovery Rate (FDR) method to control for type I errors. Additionally, the Impact value, derived from the pathway topology analysis, was calculated to quantify the overall significance of each pathway based on both enrichment and structural importance.
