# Supplementary material for: Exploration of the Possible Relationships Between Gut and Hypothalamic Inflammation and Allopregnanolone: Preclinical Findings in a Post-Finasteride Rat Model
Source: Biomolecules. 2025 Jul 18;15(7):1044. doi: 10.3390/biom15071044 (PMC12293867; doi:10.3390/biom15071044)

M = Marker
C = Control group
F = Finasteride-treated group
A = Finasteride + Allopregnanolone-treated group

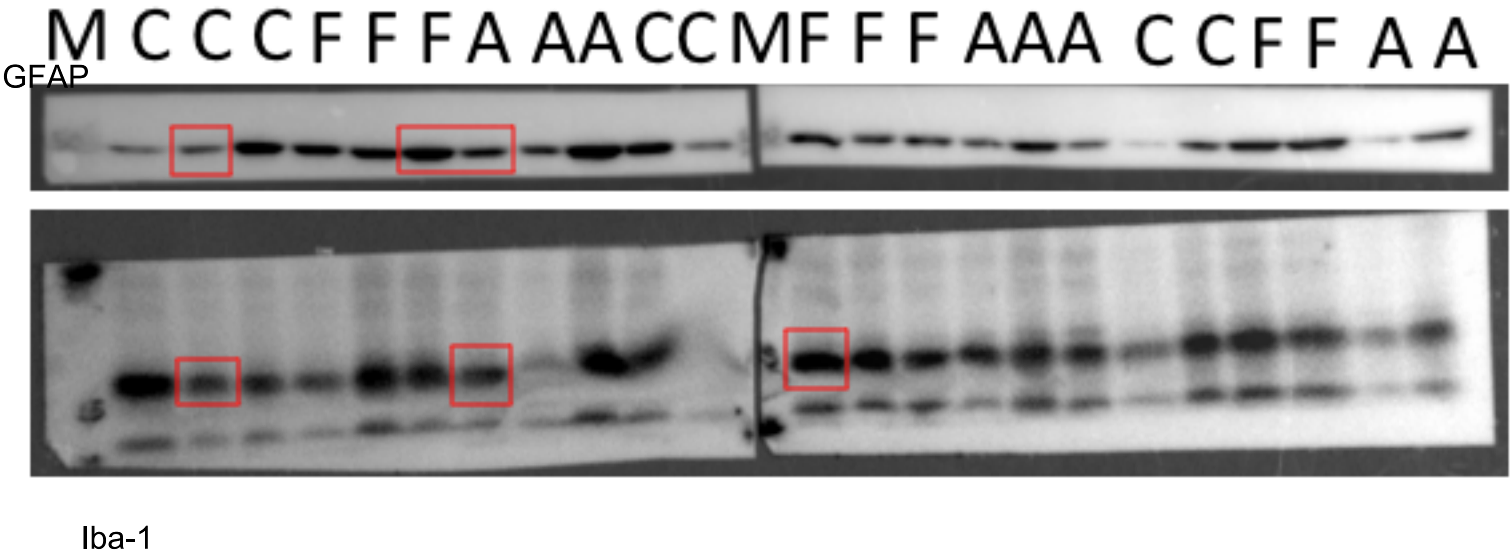

Supplement: Supplementary file 1 [file biomolecules-15-01044-s001.zip › biomolecules-3641773/original WB figure/Blots 2 with frame.pdf]
